# Porcine Bile Acids Improve Antioxidant Status and Immune Function by Increasing *Hungatella* Abundance with Different Protein Level Diets in Late-Laying Hens

**DOI:** 10.3390/ani15040500

**Published:** 2025-02-10

**Authors:** Ronghui Xing, Pengfei Du, Ziyang Wang, Zongze Fan, Longfei Wang, Yanqun Huang, Wen Chen, Xuemeng Si

**Affiliations:** Institute of Animal Science and Technology, Henan Agricultural University, Zhengzhou 450046, China; xrh0620@163.com (R.X.); duke17839966195@163.com (P.D.); wangziyang66@outlook.com (Z.W.); fanzongze0927@outlook.com (Z.F.); wlongfei2024@outlook.com (L.W.); hyanqun@aliyun.com (Y.H.); cchenwen@henau.edu.cn (W.C.)

**Keywords:** bile acids, low-protein diets, antioxidant status, immune function, *Hungatella*

## Abstract

The modern livestock industry faces significant challenges due to the extension of production cycles and a growing shortage of feed resources. Low-protein (LP) diets offer potential environmental advantages whereas damage intestinal integrity and increase pro-inflammatory cytokines. Bile acids (BAs), metabolic products of cholesterol in the liver, possess strong antioxidative and immune properties. This study evaluated the effects of dietary porcine BA supplementation at different protein levels on the antioxidant status, immune function, and gut microbiota of late-laying hens. A 2 × 2 factorial design was employed with 198 Hy-Line Brown laying hens (62 weeks old), randomly divided into one of four treatment groups with 8 replicates for each treatment. Diets included normal (16.42%) or low-protein (15.35%) levels, with porcine BAs supplemented at 0 or 120 mg/kg (62–69 weeks) and 0 or 200 mg/kg (70–75 weeks) over 14 weeks. Dietary protein levels and bile acids had no effect on organ development in laying hens. Low-protein diets increased serum anti-inflammatory cytokines IL-4 and IL-10 but elevated pro-inflammatory cytokines IL-1β (*p* < 0.05), corroborated by higher *IL-10* gene expression in the ileum mucosa (*p* < 0.05). The supplementation of bile acids improved immune function by increasing serum IL-4 and TGF-β while decreasing IL-6 levels (*p* < 0.05). Moreover, bile acids treatments upregulated *IL-10*, *TGF-β* and *ZO-1* gene expression in the ileum mucosa (*p* < 0.05) and alleviated oxidative stress by enhancing serum GSH-Px activity (*p* < 0.05). Additionally, both low-protein diets and bile acids enriched beneficial cecal bacteria, including *Hungatella* and *Blautia*, primarily linked to immune function. These findings demonstrate that porcine bile acids administration improve antioxidant capacity, immune function and gut microbiota dysbiosis. These beneficial effects were associated with *Hungatella* and *Blautia* levels, which may be responsible for the porcine bile acids efficacy in poultry nutrition.

## Simple Summary

In the current study, the effects of dietary bile acids on the antioxidant status, immune function, and gut microbiota with different crude protein levels diets in late-laying hens were evaluated. The outcome showed that dietary supplemented with bile acids improved antioxidant capacity, immune function and gut microbiota dysbiosis. These positive effects were associated with the increasing abundance of beneficial bacteria, which may be responsible for the bile acids efficacy in poultry nutrition.

## 1. Introduction

The modern livestock industry faces significant challenges due to the extension of production cycles and a growing shortage of feed resources. Low-protein (LP) diets have received considerable attention as a resource conservation strategy, offering potential environmental advantages such as reduced production costs and decreased nitrogen emissions [1]. However, the long-term adoption of LP diets to conserve protein feed resources may compromise the production performance of laying hens. Studies have reported that LP diets can damage intestinal integrity and increase pro-inflammatory cytokines [2,3]. Meanwhile, as the laying cycle lengthens, late-phase laying hens experience age-related declines in antioxidant capacity, leading to free radical accumulation and homeostatic imbalances [4,5]. Free radical-induced oxidative stress triggers chain reactions, causing varying degrees of damage, including protein structural disruption and lipid peroxidation [6]. Therefore, identifying feed additives that enhance the nutritional efficacy of LP diets is essential for extending the production cycle and improving laying performance.

Bile acids (BAs), metabolic products of cholesterol in the liver, are major components of bile in humans and animals [7]. Primary BAs are synthesized in the liver and converted into secondary BAs in the intestine through microbial activity [8,9]. Bile acids and microbiota share a close relationship and can regulate one another [10]. Li reported that bile acids enhanced intestinal health in hens on a high-fat diet by increasing the abundance of beneficial bacteria (e.g., *Firmicutes*) and decreasing the abundance of harmful bacteria (e.g., *Actinomycetes*) [11]. Furthermore, it has been demonstrated that intestinal microbiota regulates the metabolism of bile acids through bile salt hydrolase (BSH), 7-dehydroxylation, hydroxysteroid dehydrogenase (HSDH) or amide conjugation reactions [12]. Moreover, BAs play a crucial role in intestinal immunity by inhibiting pathogenic bacteria and maintaining intestinal homeostasis [13,14,15]. For instance, ursodeoxycholic acid (UDCA) alleviated intestinal inflammation in mice [16], while cholic acid (CA) and goose deoxycholic acid (CDCA) inhibited the proliferation of pathogenic bacteria such as *Staphylococcus aureus* [17]. Additionally, BAs, as endogenous molecules, possess strong antioxidative properties [18]. Dietary BA administration has been shown to enhance the antioxidant capacity of piglets and alleviate heat stress in broilers [19,20], highlighting their potential as nutritional additives.

However, a limited number of studies have explored the effects of exogenous BAs on the antioxidant capacity, immune function and gut microbiota of laying hens under varying crude protein levels. Therefore, this experiment aims to investigate the impact of BA supplementation in LP diets on the antioxidant capacity, immune response, and gut microbiota of laying hens. The findings may provide novel strategies to prolong productive lifespans and mitigate performance declines during the late-laying period.

## 2. Materials and Methods

### 2.1. Animals and Experimental Design

In this trial, a total of 192 Hy-line Brown layers (62 weeks of age) were selected in Feng yuan Poultry Co., Ltd. (Nanyang, China) and randomly allocated to the basal diet group (CON), the basal diet with 120 mg/kg (62–69 weeks) and 200 mg/kg (70–75 weeks) BAs group (CON + BA), as well as the LP diet group (LP) and the LP diet with 120 mg/kg (62–69 weeks) and 200 mg/kg (70–75 weeks) BAs group (LP + BA), arranged in a 2 × 2 factorial experimental design with each treatment including 8 replicates of 6 hens. All hens were housed in 3-layer vertical cages located in a temperature-controlled room with a lighting schedule of 16 h of light and 8 h of darkness. The ambient temperature was 23 to 25 °C with 30% to 50% relative humidity. Feed and water were freely available during the whole experiment. The BA products used in the trial were supplied by the Henan 91 Chinese Medicine Research Institute Co., Ltd. (Zhengzhou, China) and derived from the gallbladders of pigs, which were more readily available and inexpensive compared with other sources of BAs. The composition of the BAs was determined by liquid chromatography–tandem mass spectrometry [21] and consisted of 7.73% hyocholic acid, 68.31% hyodeoxycholic acid and 18.96% chenodeoxycholic acid. The dosage of BA supplementation was adjusted based on previous studies [22,23,24]. The experimental period lasted from 62 to 75 weeks of age. The composition of the basal normal or LP diets is shown in Table 1; metabolizable energy and the selected essential amino acids in the diets were formulated to meet the requirements recommended by the Hy-Line Brown Management Guide (2018) [25]. All diet samples were ground to pass through a 0.5 mm screen; these samples were dried overnight at 105 °C and ashed at 600 °C for 2 h for an analysis of ash content. Samples of the diet were analyzed for crude protein following the procedures of method 990.9 [26]. In addition, the calcium and phosphorus contents of feeds were determined through ethylenediaminetetraacetic acid titration and ammonium metavanadate colorimetry, respectively.

### 2.2. Sample Collection

On the last day of the experiment (the 98th day), one bird was randomly selected per replicate (8 birds per treatment) for blood collection after a 12 h fast. Sub-wing vein blood was centrifuged in a centrifuge at 1500× *g* in 4 °C for 10 min to obtain serum. After sacrificing by cervical dislocation, the heart, liver, spleen and pancreas were removed and weighted for determining the organ index. The cecal chyme and ileum mucosa were taken out from hens, quick-frozen in liquid nitrogen and stored at −80 °C.

### 2.3. Determination of the Organ Index

The organ index (%) was calculated as the organ fresh weight (kg)/hen weight (kg) × 100% before slaughter.

### 2.4. Serum Biochemical Parameters

Serum parameters, including interleukin-4 (IL-4), interleukin-10 (IL-10), tumor necrosis factor-β (TNF-β), interleukin-1α (IL-1α), interleukin-1β (IL-1β), interleukin-6 (IL-6), interleukin-17α (IL-17α), tumor necrosis factor-α (TNF-α), interferon-γ (IFN-γ), diamine oxidase (DAO) activity and D-lactic acid (D-LA) content in serum were determined using ELISA kits (Jiangsu Meibiao Biological Technology, Taizhou, China) following the manufacturer’s protocols.

### 2.5. Antioxidant Enzyme Activity Assay

The activities of superoxide dismutase (SOD), glutathione peroxidase (GSH-Px), catalase (CAT), total antioxidant capacity (T-AOC), the content malondialdehyde (MDA) and hydrogen peroxide (H_2_O_2_) in serum were analyzed using reagent kits (Jiancheng, Bioengineering Institute, Nanjing, China).

### 2.6. Total Community DNA Extraction

Microbial DNA in the cecal samples was extracted using the QIAamp Fast DNA Stool Mini Kit (Qiagen, Hilden, Germany) according to the instructions. Microbial community DNA was examined using NanoDrop 2000 spectrophotometer (Thermo Scientific, Wilmington, DE, USA) and 1% agarose gel electrophoresis to assess the quantity and quality.

### 2.7. 16S rRNA Gene Amplicon Sequencing

The V3-V4 region of bacteria was amplified with 338 F (5′-ACTCCTACGGGAGGCAGCA-3′) and 806 R (5′-GGACTACHVGGGTWTCTAAT-3′) amplification primers. The PCR amplification program included: 4 µL 5 × FastPfu buffer, 2 µL 2.5 mM dNTPs, 0.8 µL 5 µM each primer, 0.4 µL FasPfu Polymerase and 10 ng template DNA. The obtained PCR products were purified using the AxyPrep DNA Gel Extraction Kit (Axygen Biosciences, Union City, CA, USA). Samples were sequenced on Illumina MiSeq PE300 platform (Illumina, San Diego, CA, USA) by Shanghai Personal Biotechnology Technology Co., Ltd. (Shanghai, China).

The raw sequencing reads were processed for filtering and demultiplexing using Vsearch (version 2.17.0). High-quality circular consensus sequencing (CCS) reads were retained by setting the parameters to minPasses ≥ 5 and minPredictedAccuracy ≥ 0.9. Sample assignment of CCS reads was conducted based on barcode sequences using Cutadapt (version 2.7), ensuring accurate demultiplexing. Subsequently, CCS reads without primers or falling outside the length range of 1200–1650 bp were discarded using primer identification and length-filtering functionalities in Cutadapt. Chimera sequences were detected and removed using the de novo algorithm of Vsearch uchime to ensure data quality and eliminate potential artifacts. After obtaining clean reads, sequences with a similarity of 97% or higher were clustered into operational taxonomic units (OTUs) using Vsearch (version 2.17.0). Low-abundance OTUs, defined as those with fewer than 2 counts across all samples, were filtered out to exclude noise and ensure reliable downstream analyses. Subsequently, the representative OTUs were classified into organisms by a naive Bayesian model using an RDP classifier (version 2.2) based on the Greengenes database (version 13.8), with a confidence threshold value of 0.8.

### 2.8. Gene Expression by Real-Time PCR

Total RNA was isolated from the ileum using a total RNA extraction kit (TransGen Biotech Co., Ltd., Beijing, China) in accordance with the manufacturer’s instructions. The quality and concentration of extracted RNA were determined by agarose gel electrophoresis and nucleic acid quantification, respectively. The cDNA was synthesized by the cDNA reverse transcription kit (Takara, Dalian, China). The obtained cDNA was used for gene expression analysis using SYBR green qPCR master mix (Takara, Dalian, China). The amplification conditions are as follows: 95 °C for 15 s, followed by 40 cycles of 95 °C for 30 s and 60 °C for 34 s, with a final melting curve analysis. The specific primers were designed online (https://www.ncbi.nlm.nih.gov (accessed on 9 September 2024)). The β-actin was used as a housekeeping gene to normalize target gene expression. Primers are presented in Table 2.

### 2.9. Statistical Analyses

Data were analyzed by ANOVA in a 2 × 2 factorial design using the GLM procedures of SPSS 23.0 (SPSS Inc., Chicago, IL, USA). The main effects (BA or LP) and interactions between the 2 factors were carried out. A Tukey’s test was applied when any of the interactions showed significance. Each replicate was the experimental unit. Data were shown as the means and pooled standard errors (SEMs). The results were considered significantly different at *p* < 0.05.

The community composition was visualized using stacked bar plots generated with the ggplot2 package (version 2.2.1) in R. A correlation network was constructed based on correlation coefficients using the igraph package (version 1.1.2) in R. Between-group Venn analyses were performed with the Venn diagram package (version 1.6.16) in R, and unique and shared species or OTUs were identified using UpSet plots created with the UpSet R package (version 1.3.3). The comparison of species among groups was conducted using the Kruskal–Wallis H test to assess differences in microbial abundance between groups, followed by a Tukey’s HSD test for pairwise comparisons to identify specific taxa with significant differences, implemented in the vegan package (version 2.5.3) in R. Alpha diversity indices, including Chao1, Shannon, Simpson and Pielou, were calculated using QIIME (version 1.9.1) by a Kruskal–Wallis H test. The Bray–Curtis distance matrix was computed in R using the vegan package (version 2.5.3). A principal coordinates analysis (PCoA) of the Bray–Curtis distances was also performed using the vegan package and visualized with ggplot2 (version 2.2.1). The Spearman correlation coefficients of microbial taxa were calculated and visualized using the heatmap package (version 2.3.1) in R, with statistical significance set at *p* < 0.05.

## 3. Results

### 3.1. Organ Index

The effects of bile acids and dietary protein levels on the organ index are presented in Table 3. The results indicated no significant differences in the organ index, but a decreasing trend in the spleen index was observed in low-protein diets (*p* = 0.053). No significant interactions between bile acids and dietary protein levels were observed.

### 3.2. Serum Inflammatory Cytokines and Ileal-Immunity-Related Gene mRNA Expression

**Figure 1:**
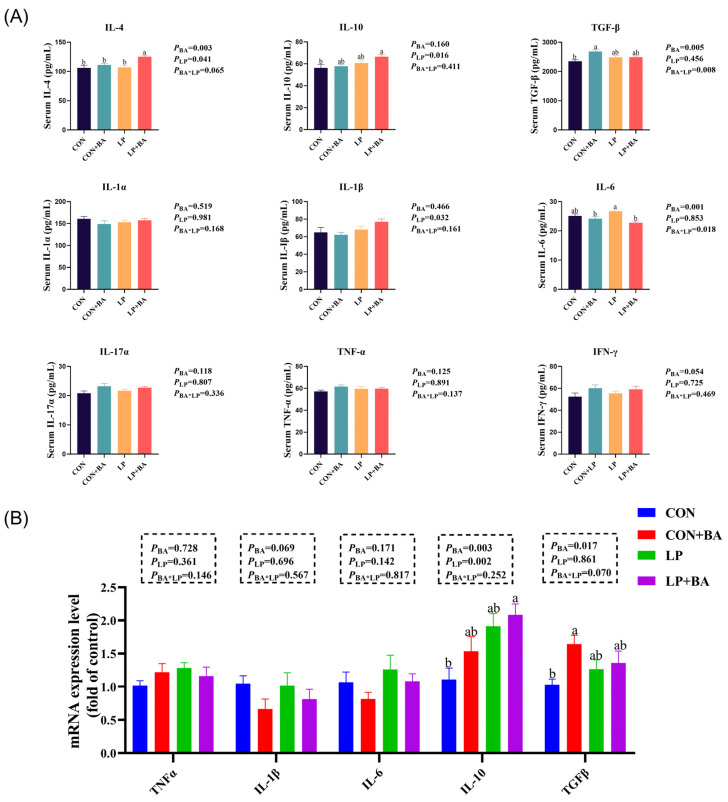
Serum inflammatory cytokines and ileal-immunity-related gene mRNA expression. (**A**) Serum inflammatory cytokines of laying hens. (**B**) mRNA levels of inflammatory cytokines were detected by qRT-PCR in the ileum. Each value represents the mean value of 8 replicates per treatment (*n* = 8). Different superscripts in bars indicate a significance difference (*p* < 0.05). BAs, bile acids. LP, low-protein. IL-4, interleukin-4. IL-10, interleukin-10. TNF-β, tumor necrosis factor-β. IL-1α, interleu-kin-1α. IL-1β, interleukin-1β. IL-6, interleukin-6. IL-17α, interleukin-17α. TNF-α, tumor necrosis factor-α. IFN-γ, interferon-γ. CON, basal diet CON + BA, basal diet + 120 mg/kg (62–69 weeks) and 200 mg/kg (70–75 weeks) BA. LP, low-protein diet. LP + BA, LP diet + 120 mg/kg (62–69 weeks) and 200 mg/kg (70–75 weeks) BA.

The immune function is shown in Figure 1. LP diets increased IL-4 (*p* = 0.041), IL-10 (*p* = 0.016) and IL-1β (*p* = 0.032) in serum (Figure 1A). The supplementation of BAs increased serum IL-4 (*p* = 0.003) and TGFβ (*p* = 0.005) but decreased IL-6 (*p* = 0.001; Figure 1A). Both LP diets and BAs upregulated ileum IL-10 expression (*P*_LP_ = 0.002, *P*_BA_ = 0.003; Figure 1B). Moreover, BA treatments increased gene TGFβ (*p* = 0.017) expression in the ileum (Figure 1B). No significant interactions between the 2 dietary factors were observed.

### 3.3. Antioxidant Status

**Figure 2:**
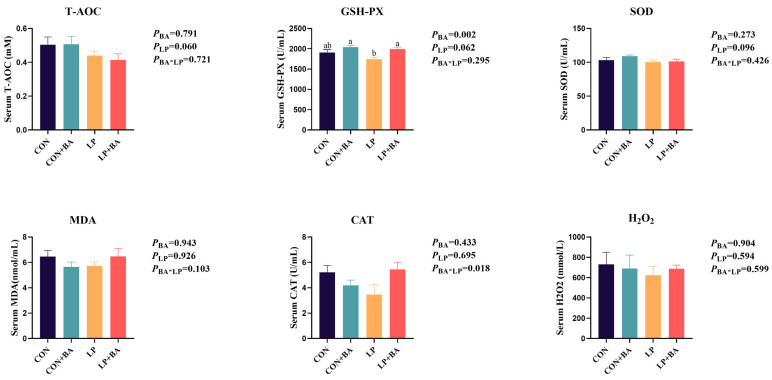
Antioxidant status. Each value represents the mean value of 8 replicates per treatment (*n* = 8). Different superscripts in bars indicate a significance difference (*p* < 0.05) and trends *(p* > 0.05 and ≤ 0.1). BAs, bile acids. LP, low-protein. SOD, superoxide dismutase. GSH-Px, glutathione peroxidase. CAT, Catalase. T-AOC, total antioxidant capacity. MDA, malondialdehyde. H_2_O_2_, hydrogen peroxide. CON, basal diet. CON + BA, basal diet + 120 mg/kg (62–69 weeks) and 200 mg/kg (70–75 weeks) BA. LP, low-protein diet. LP + BA, LP diet + 120 mg/kg (62–69 weeks) and 200 mg/kg (70–75 weeks) BA.

Figure 2 shows the effects of bile acids and dietary protein levels on the serum antioxidant enzyme activities of late-laying hens. The supplementation of BAs increased serum GSH-Px activity (*p* = 0.002). LP diets had a trend of decreasing the serum T-AOC (*p* = 0.060) and GSH-Px (*p* = 0.062). No significant interactions between the two dietary factors were observed.

### 3.4. Serum Intestinal Permeability Biomarkers and Ileal-Barrier-Related Gene mRNA Expression

**Figure 3:**
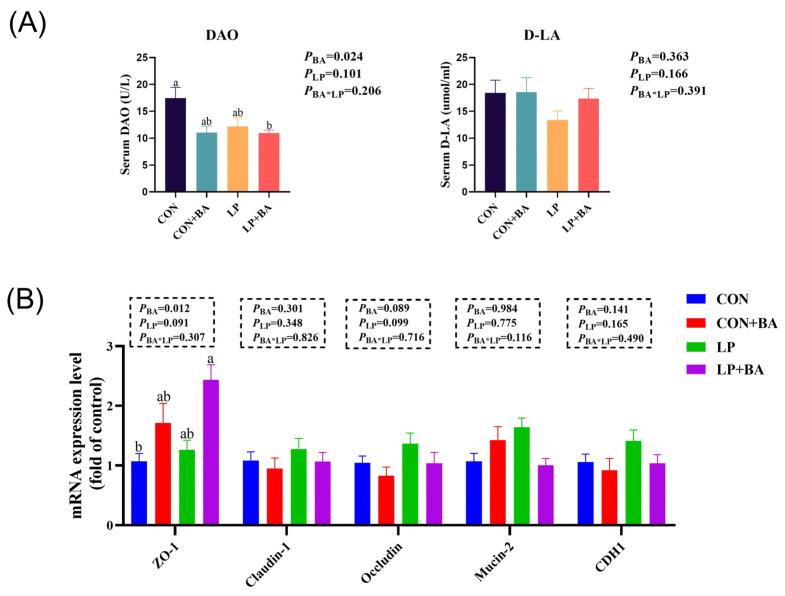
Serum intestinal permeability biomarkers and ileal-barrier-related gene mRNA expression. (**A**) Serum diamine oxidase activity and D-lactic acid content (**B**) mRNA levels of intestinal barrier were detected by qRT-PCR in the ileum. Each value represents the mean value of 8 replicates per treatment (*n* = 8). Different superscripts in bars indicate a significance difference (*p* < 0.05). BA, bile acid. LP, low-protein. DAO, diamine oxidase. D-LA, D-lactic acid. ZO-1, tight junction protein 1. Claudin-1, claudin-1. Occludin, occludin. Mucin-2, mucin 2. CDH1, cadherin 1. CON, basal diet. CON + BA, basal diet + 120 mg/kg (62–69 weeks) and 200 mg/kg (70–75 weeks) BA. LP, low-protein diet. LP + BA, LP diet + 120 mg/kg (62–69 weeks) and 200 mg/kg (70–75 weeks) BA.

The effects of bile acids and dietary protein levels on serum intestinal permeability biomarkers and ileal-barrier-related gene mRNA expression are shown in Figure 3. The supplementation of BAs had no effect on serum D-LA content whereas could decrease serum DAO activity (*p* = 0.024) (Figure 3A). BA treatments increased ileum ZO-1 (*p* = 0.012) expression (Figure 3B). No significant interactions between the two dietary factors were observed.

### 3.5. Cecal Microbiota

**Figure 4:**
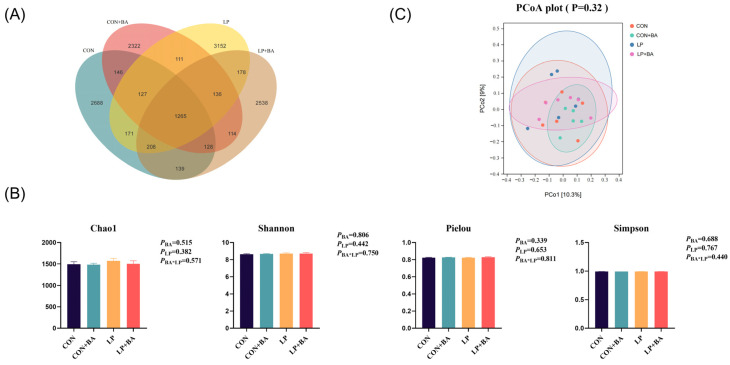
The alpha and beta diversity of cecal microbiota. (**A**) Venn diagrams for bacterial OTUs (operational taxonomic units). (**B**) Alpha diversity. (**C**) A principal coordinate analysis (PCoA) based on the Braye-Curtis distance at the OTU level. Each value represents the mean value of 6 replicates per treatment (*n* = 6). BA, bile acid. LP, low-protein. CON, basal diet. CON + BA, basal diet + 120 mg/kg (62–69 weeks) and 200 mg/kg (70–75 weeks) BA. LP, low-protein diet. LP + BA, LP diet + 120 mg/kg (62–69 weeks) and 200 mg/kg (70–75 weeks) BA.

**Figure 5:**
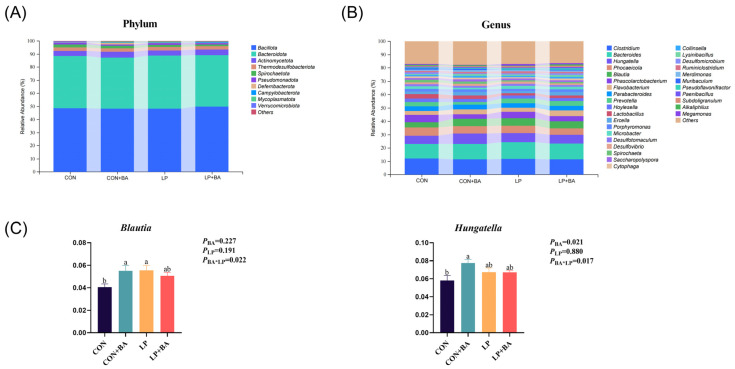
The differences in taxonomic abundance among groups. (**A**) Taxonomic composition of the cecal microbiota at the phylum. (**B**) Taxonomic composition of the cecal microbiota at the genus. (**C**) Relative abundance of cecal microbiota at the genus level with significant changes and specific functions. Each value represents the mean value of 6 replicates per treatment (*n* = 6). Different superscripts in bars indicate a significance difference (*p* < 0.05). BA, bile acid. LP, low-protein. CON, basal diet. CON + BA, basal diet + 120 mg/kg (62–69 weeks) and 200 mg/kg (70–75 weeks) BA. LP, low-protein diet. LP + BA, LP diet + 120 mg/kg (62–69 weeks) and 200 mg/kg (70–75 weeks) BA.

The cecal microbiota composition is shown in Figure 4 and Figure 5. A total of 13,741 OTUs were obtained from the samples. The CON group had 2688 special OTUs, the CON + BA group had 2322 special OTUs, the LP group had 3152 special OTUs and the LP + BA group had 2538 special OTUs (Figure 4A). The diversity of the microbial communities was measured by the Chao1 diversity index, Shannon diversity index, Pielou diversity index and Simpson diversity index (Figure 4B). The dietary treatment did not affect both the Chao1, Shannon, Pielou and Simpson diversity indexes (*p* > 0.05). PCoA showed that all groups have the small degree of dispersion (Figure 4C). The taxonomic abundance is displayed. At the phylum level, the main populations were *Bacillota* and *Bacteroidetes*, followed by *Actinomycetoma* and *Themode sulfobacteriota* (Figure 5A). At the genus level, *Clostridium*, *Bacteroides*, *Hungatella*, *Phocaeicola*, *Blautia* and *Phascolarctobacterium* were dominant (Figure 5B). An interaction was found between BAs and dietary protein levels in *Blautia* (*p* = 0.022) in which the LP diet group and the normal protein diet with BA supplementation group had a higher *Blautia* abundance compared with the other groups (Figure 5C). A significant interaction (*p* = 0.017) between BAs and dietary protein levels in *Hungatella* was observed; birds fed the normal protein diet with BA supplementations emerged with the highest *Hungatella* abundance compared to those fed any other dietary treatments (Figure 5C).

### 3.6. Correlations Between Cecal Microbiota and Serum Indicators

**Figure 6:**
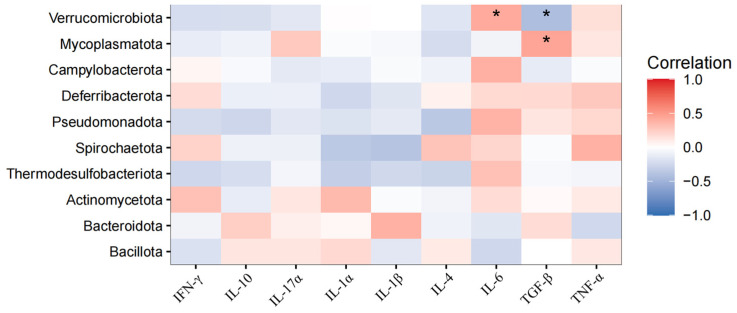
Correlations between cecal microbiota and serum indicators. Horizontal for serum inflammatory cytokines, vertical for the top 10 abundant microbiota of phylum. Red for positive correlation blue for negative correlation. *, 0.01 < *p* < 0.05.

**Figure 7:**
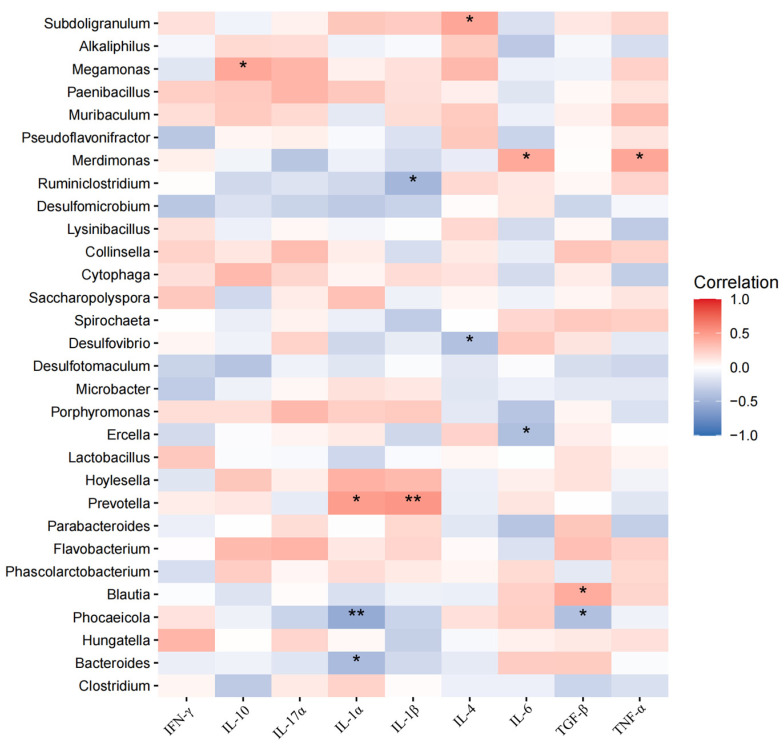
Correlations between cecal microbiota and serum indicators. Horizontal for serum inflammatory cytokines, vertical for the top 30 abundant microbiota of genus. Red for positive correlation, blue for negative correlation. *, 0.01 < *p* < 0.05; **, 0.001 < *p* < 0.01.

A Spearman coefficient was used to evaluate the correlation between cecal microbiota and serum indicators. We selected the top 10 abundant microbiota of phylum and the top 30 abundant microbiota of genus to make correlations with specific indicators. According to Figure 6, at the phylum level, *Verrucomicrobiota* was positively correlated with serum IL-6 (*p* < 0.05) and negatively correlated with TGFβ (*p* < 0.05). *Mycoplasmatota* was positively correlated with TGFβ (*p* < 0.05). According to Figure 7, at the genus level, *Megamonas*, *Subdoligranulum* and *Blautia* were positively correlated with serum anti-inflammatory cytokines (IL-10, IL-4, or TGFβ) (*p* < 0.05) and *Desulfovibrio* and *Phocaeicola* were negatively correlated with anti-inflammatory cytokines (IL-4 or TGFβ) (*p* < 0.05). *Prevotella* and *Merdimonas* were positively correlated with serum pro-inflammatory cytokines (IL-1α, IL-11β, IL-6, or TGFα) (*p* < 0.05), while *Phocaeicola*, *Bacteroides*, *Ruminiclostridium* and *Ercella* were negatively correlated with serum pro-inflammatory cytokines (IL-1α, IL-11β and IL-6) (*p* < 0.05).

## 4. Discussion

This study investigated the effects of dietary BA supplementations on the antioxidant status, immune function and gut microbiota of laying hens under different crude protein levels. The findings provide major insights into the multifaceted role of BAs in poultry nutrition. Relative organ weights are widely used to assess the nutritional and developmental status of livestock and poultry [27,28]. Consistent with previous findings [22], this study showed that neither LP diets nor BAs affected organ development in late-laying hens. These results indicated that LP diets may meet the basic nutritional needs of growth and development in late-laying hens.

Cytokines, as critical modulators of intestinal inflammation, play a central role in mediating the immune response [29]. Although previous studies have reported that LP diets increased pro-inflammatory cytokines [2,3], our results demonstrated a more complex response, with LP diets elevating both pro-inflammatory cytokine (IL-1β) and anti-inflammatory cytokines (IL-4 and IL-10) in serum. This dual effect may suggest a compensatory mechanism where the upregulation of pro-inflammatory cytokines stimulates as a subsequent anti-inflammatory response to maintain immune homeostasis. This hypothesis aligns with the observation of increasing ileal IL-10 expression in the LP diet group, yet further research is needed to confirm underlying mechanisms. Furthermore, the anti-inflammatory properties of BAs are well documented [30,31]. Similar results were obtained in our study, where BAs increased serum IL-4 and TGFβ and decreased IL-6. This result was also supported by the quantification of ileal mucosa, and both BA and LP diets upregulated the expression of anti-inflammatory cytokines in the ileal. These results collectively highlight the complementary effects of BAs in mitigating the immune stress induced by LP diets.

As laying hens age, their antioxidative capacity declines, resulting in free radical accumulation and oxidative damage [4,5]. Total antioxidant capacity is the ability of the organism to resist oxidative damage [32]. Enzymes in the glutathione-dependent antioxidant system, including SOD, GSH-Px and CAT, play crucial roles in scavenging reactive oxygen species (ROS) and protecting cells from oxidative damage [33,34]. Our study confirmed that BA supplementation enhanced serum GSH-Px activity, which is consistent with previous findings for broilers and piglets [19,20]. This may imply that BAs alleviated oxidative stress to some extent in late-laying hens by improving their antioxidant defense systems.

The gut mucosal system, as the largest mucosal immune organ, is essential for maintaining intestinal barrier function and preventing pathogen infiltration [35]. In the study, we observed that BA supplementation decreased serum DAO activity, an indicator of intestinal damage [36]. Furthermore, the upregulation of *ZO-1* expression in the ileal mucosa supports the hypothesis that BAs enhance tight junction integrity, thereby improving barrier function. These results are in line with previous evidence demonstrating the protective effects on the intestinal barrier [37,38]. Importantly, this protective effect in the intestinal barrier may also contribute to the observed improvements in immune and antioxidant responses, as an intact epithelial barrier reduces the risk of systemic inflammation and oxidative stress.

The interactions between BAs and gut microbiota further underscore the multifaceted role of BAs in poultry nutrition. While early studies [23] and our findings indicate that BA supplementation does not alter the diversity of cecum microbiota, BAs selectively influence microbial composition. Notably, the abundance of *Blautia* and *Hungatella* enhanced significantly with BA supplementation. *Blautia*, a genus of commensal specialized anaerobes, has been shown to promote intestinal regulatory T cells and the production of short-chain fatty acids, which played an important role in maintaining homeostasis and preventing inflammation [39,40]. The increase in *Blautia* abundance observed in this study may explain the elevated levels of serum anti-inflammatory cytokines, including IL-10 and TGF-β. Similarly, *Hungatella*, which degrades glycosaminoglycans into substrates beneficial for gut health [41,42], was also enriched in the BA administration. Costa et al. [43] reported that the relative abundance of *Hungatella* was negatively correlated with dietary inflammatory indices in individuals with intestinal constipation. This suggests that BAs contribute to maintaining the stability of the intestinal flora and inhibit intestinal inflammation by selectively promoting beneficial microbial populations.

The serum biochemical index reflects the physiological health status of the body, which is closely related to the intestinal microbiota. *Verrucomicrobiota* is widely distributed in the human and animal gut and plays an important role in host gut health. *Akkermansia muciniphila*, a representative genus of *Verrucomicrobiota*, has been identified as being linked to inflammation response [44]. *Akkermansia muciniphila* may trigger an inflammatory response by activating the NLRP6 inflammasome [45]. *Prevotella* is closely related to the occurrence of mucosal inflammation and metabolic disorders [46]. It has been reported that *Prevotella* contributes to inflammation by increasing metabolites (e.g., propionic acid) and activating pro-inflammatory signaling pathways (e.g., TLR2 signaling pathways) [42]. *Phocaeicola dorei*, an important species of the genus *Phocaeicola*, has been proven to promote anti-inflammatory responses by producing short-chain fatty acids (e.g., butyric acid) and inhibiting the production of pro-inflammatory cytokines (e.g., IL-6 and TNF-α) [47]. In short, the microbiota is closely linked to host health, and these microbial shifts highlight the potential of BAs to modulate gut microbiota composition in ways that favor intestinal health and immunity.

## 5. Conclusions

In summary, this study reveals that LP diets have no adverse effects on organ development in late-stage laying hens but may compromise intestinal integrity by increasing inflammatory cytokines. Conversely, BA supplementation improves the antioxidant capacity, intestinal barrier function, and immune responses by modulating the gut microbiota. These findings indicate that dietary BAs provide a promising strategy to mitigate the negative effects of LP diets and prolong the productive lifespan of laying hens. The synergistic effects of LP diets and BAs on immunity and gut health want further investigation in order to optimize their application to poultry nutrition.

## Figures and Tables

**Table 1:** Ingredients and nutrient content of diets (%, air-dry basis).

Item	Normal Protein	Low Protein
ingredients		
Corn	65.75	67.30
Soybean meal	22.00	18.37
Bran	-	1.90
Liquid methionine	0.16	0.16
Stone powder	9.60	9.60
CaHPO_4_	0.80	0.80
Choline	0.14	0.14
Premix ^1^	1.50	1.50
L-lysine·HCl, 78.6%	-	0.10
DL-methionine, 99%	0.05	0.08
L-threonine, 99%	-	0.05
Total	100	100
Calculated nutrient content		
ME, KJ/kg	2650	2650
Lys	0.77	0.74
Met	0.36	0.36
Thr	0.57	0.55
Assayed nutrient content		
Crude protein	16.42	15.35
Calcium	3.59	4.01
Total phosphorus	0.44	0.40
Crude ash	14.00	15.08

^1^ Premix provided the following per kilogram of the diet: vitamin A, 4500 IU; vitamin D, 5500 IU; vitamin E, 16 IU; vitamin K, 0.5 mg; thiamine, 2.0 mg; riboflavin, 5.0 mg; pyridoxine, 4.5 mg; vitamin, B_12_, 24 mg; Cu (CuSO_4_·5H_2_O), 14 mg; Fe (FeSO_4_·7H_2_O), 85 mg; Zn (ZnSO_4_·7H_2_O), 75 mg; Mn (MnSO_4_·H_2_O), 78 mg; Se (NaSeO_3_), 0.7 mg; I (KI), 0.7 mg; calcium pantothenate, 15.0 mg; folate, 2.5 mg; biotin, 0.15 mg; nicotinic acid 42 mg. ME, apparent metabolism energy.

**Table 2:** Gene-specific primers for real-time quantitative reverse transcription PCR.

Gene ID ^#^	Gene	Primer Sequences (5′→3′)	Product Length, bp
XM_046925214.1	*ZO-1*	F: GAAGAGAGCACAGAACGCAG	123
		R: CACTTGTGGCAAGCTGAAGT	
NM_001013611.2	*Claudin-1*	F: TCTGGTGTTAACGGGTGTGA	117
		R: GTCTTTGGTGGCGTGATCTT	
NM_205128.1	*Occludin*	F: CGTTCTTCACCCACTCCTCC	107
		R: CCAGAAGACGCGCAGTAAGA	
NM_001039258.3	*CDH1*	F: AGCCAAGGGCCTGGATTATG	157
		R: GATAGGGGGCACGAAGACAG	
NM-001318434.1	*Mucin-2*	F: AGTGGCCATGGTTTCTTGTC	80
		R: TGCCAGCCTTTTTATGCTCT	
NM_204628.1	*IL-6*	F: CCCTCACGGTCTTCTCCATA	100
		R: CTCCTCGCCAATCTGAAGTC	
NM_204267.1	*TNFα*	F: ACTGGGCGGTCATAGAACAG	120
		R: AGATGGGAAGGGAATGAACC	
NM_001318456.1	*TGFβ*	F:GCTCTTTGGCCCAATACTCA	88
		R:CTGTACAACAGCACCCAGGA	
NM_001004414.4	*IL-10*	F: GCTCTGAGCACAGTCGTTTG	154
		R: CAGATGGGGACGTGGTTACG	
NM_204524.2	*IL-1β*	F: CTGCCTGCAGAAGAAGCCT	135
		R: CGCAGCAGTTTGGTCATGG	
NM_205518.1	*β-actin*	F: GTCCACCGCAAATGCTTCTAA	78
		R: TGCGCATTTATGGGTTTTGTT	

^#^ ZO-1, tight junction protein 1; Claudin-1, claudin-1; Occludin, occludin; CDH1, cadherin 1; Mucin-2, mucin 2; IL-6, interleukin-6; TNF-α, tumor necrosis factor-α; TNF-β, tumor necrosis factor-β; IL-10, interleukin-10; IL-1β, interleukin-1β; β-actin, reference genes.

**Table 3:** Organ index ^1^.

Item			Heart %	Liver %	Spleen %	Pancreas %
	BA	LP				
CON	−	−	0.40	1.71	0.11	0.17
CON + BA	+	−	0.39	1.76	0.12	0.16
LP	−	+	0.39	1.69	0.10	0.16
LP + BA	+	+	0.35	1.61	0.11	0.16
SEM			0.008	0.025	0.003	0.003
BA factor						
−BA			0.39	1.70	0.10	0.17
+BA			0.37	1.68	0.11	0.16
LP factor						
CON			0.39	1.73	0.11	0.16
LP			0.37	1.65	0.10	0.16
*p*-value						
BA			0.104	0.670	0.201	0.252
LP			0.122	0.101	0.053	0.564
Interaction			0.420	0.190	0.412	0.184

^1^ Each value represents the mean value of 8 replicates per treatment (*n* = 8). SEM, standard error of the mean. BA, bile acid. LP, low-protein. CON, basal diet. CON + BA, basal diet + 120 mg/kg (62–69 weeks) and 200 mg/kg (70–75 weeks) BA. LP, low-protein diet. LP + BA, LP diet + 120 mg/kg (62–69 weeks) and 200 mg/kg (70–75 weeks) BA.

## Data Availability

The original contributions presented in the study are included in the article; further inquiries can be directed to the corresponding author.
